# Navigating by Design: Effects of Individual Differences and Navigation Modality on Spatial Memory Acquisition

**DOI:** 10.3390/bs15070959

**Published:** 2025-07-15

**Authors:** Xianyun Liu, Yanan Zhang, Baihu Sun

**Affiliations:** 1 Faculty of Psychology, Tianjin Normal University, Tianjin 300387, China; 2300340008@stu.tjnu.edu.cn; 2 Key Research Base of Humanities and Social Sciences of the Ministry of Education, Academy of Psychology and Behavior, Tianjin Normal University, Tianjin 300387, China; 3 Tianjin Key Laboratory of Student Mental Health and Intelligence Assessment, Tianjin 300387, China

**Keywords:** spatial memory, spatial ability, navigation mode, sense of direction, virtual environment

## Abstract

Spatial memory is a critical component of spatial cognition, particularly in unfamiliar environments. As navigation systems become integral to daily life, understanding how individuals with varying spatial abilities respond to different navigation modes is increasingly important. This study employed a virtual driving environment to examine how participants with varying spatial abilities (good or poor) performed under three navigation modes, namely visual, audio, and combined audio–visual navigation modes. A total of 78 participants were divided into two groups, good sense of direction (G-SOD) and poor sense of direction (P-SOD), according to their Santa Barbara Sense of Direction (SBSOD) scores. They were randomly assigned to one of the three navigation modes (visual, audio, audio–visual). Participants followed navigation cues and simulated driving behavior to the end point twice during the learning phase, then completed the route retracing task, recognizing scenes task and recognizing the order task. Significant main effects were found for both SOD group and navigation mode, with no interaction. G-SOD participants outperformed P-SOD participants in route retracing task. Audio navigation mode led to better performance in tasks involving complex spatial decisions, such as turn intersections and recognizing the order. The accuracy of recognizing scenes did not significantly differ across SOD groups or navigation modes. These findings suggest that audio navigation mode may reduce visual distraction and support more effective spatial encoding and that individual spatial abilities influence navigation performance independently of guidance type. These findings highlight the importance of aligning navigation modalities with users’ cognitive profiles and support the development of adaptive navigation systems that accommodate individual differences in spatial ability.

## 1. Introduction

Navigation is the ability to estimate one’s position, maintain spatial orientation, and plan routes in physical space, which is fundamental to human survival. From an evolutionary standpoint, the ability to successfully navigate complex natural environments contributed directly to hunting, foraging, migration, and territorial safety (Wolbers & Hegarty, 2010). In contemporary settings, navigation remains equally crucial. It plays an indispensable role in activities such as commuting, tourism, logistics, emergency response, and military operations. Whether traversing familiar neighborhoods or exploring unfamiliar terrains, humans rely on spatial orientation to reach goals efficiently and avoid hazards.

### 1.1. Types of Spatial Knowledge

According to Siegel and White (1975), spatial knowledge can be categorized into three types, namely landmark knowledge (recognition of salient environmental features), route knowledge (sequence of turns or path-based information), and survey knowledge (global understanding of spatial layout). These knowledge types are thought to develop sequentially (Siegel & White, 1975), but some studies suggest they may also emerge in parallel (Kim & Bock, 2021; Peer et al., 2021). Since most navigation takes place in familiar or partially familiar environments, individuals need to be able to integrate environmental information and form mental representations (Golledge et al., 2000). Many studies have used these three distinct types of spatial knowledge as measures in spatial tasks.

Landmark knowledge refers to the recognition and memory of distinct features or objects in the environment—such as buildings, trees, or signs—that serve as orientation cues. These features help individuals anchor their location and monitor progress during navigation. For example, a traveler might remember passing a tall red sculpture or a convenience store on the corner (Presson & Montello, 1988).

Route knowledge involves memory for sequences of actions or turns required to move from one location to another. It may rely on encoding the spatial relationships between landmarks and directions (Kuipers, 1978). Route knowledge remains egocentric in nature, typically encoded relative to the individual’s perspective while traveling.

Survey knowledge is the most complex form, requiring the integration of spatial relationships into a mental map that supports allocentric reasoning, such as estimating distances or drawing novel shortcuts. Survey knowledge reflects a more global understanding of the environment’s layout representation or cognitive map of the environment (Siegel & White, 1975).

### 1.2. Sense of Direction and Individual Differences

Individuals may acquire spatial knowledge through direct exploration of an environment (e.g., through walking or driving) or indirectly through symbolic representations such as maps or verbal instructions (Montello et al., 2004). However, the efficiency with which this knowledge is acquired varies greatly across individuals (Burte & Montello, 2017; Kozlowski & Bryant, 1977; Wen et al., 2013). These differences are often captured by self-assessed measures of spatial ability, such as sense of direction (SOD), which reflects an individual’s confidence and perceived competence in navigating spatial environments. One widely used self-report measure is the Santa Barbara Sense of Direction (SBSOD) scale (Hegarty et al., 2002), which has shown predictive validity for spatial performance in both real and virtual environments. While SOD is a robust predictor of real-world navigation performance, it primarily captures subjective spatial orientation skills and wayfinding confidence. In contrast, spatial ability is a broader construct encompassing multiple cognitive facets, including mental rotation, spatial visualization, perspective-taking, and spatial working memory (Uttal et al., 2013). SOD does not directly measure these component skills, particularly those assessed by objective cognitive tasks. Therefore, although SOD provides insight into individual differences in navigation behavior, it does not encompass the full spectrum of spatial cognition.

Past researchers have conducted many studies on the performance of sense of direction and individual spatial ability. Prior findings suggest that individuals with a poor sense of direction (P-SOD) may struggle particularly with survey knowledge tasks, while those with a good sense of direction (G-SOD) are more likely to encode spatial layouts and build more accurate cognitive maps (Burte & Montello, 2017; Hegarty et al., 2002; Wen et al., 2013; Weisberg et al., 2014). However, G-SOD participants’ advantage over P-SOD participants in recognizing individual landmarks or recalling route order remains under debate (Wen et al., 2013).

Wen et al. (2013) divided the participants into two groups (G-SOD and P-SOD) according to their SBSOD scores and then compared participants’ performance in direction–estimation, distance–estimation, and map-sketching tasks. They found that people with a G-SOD tended to encode survey knowledge to integrate verbal and visuospatial working memory. In contrast, P-SOD participants relied on verbal working memory and lacked spatial processing, thus failed to acquire accurate survey knowledge (Wen et al., 2013). Similar findings have shown that individuals with G-SOD tend to perform better on tasks requiring survey-level understanding of the environment (Hegarty et al., 2002; Ishikawa & Montello, 2006). Interestingly, individuals who reported having a G-SOD exhibited better spatial knowledge accuracy under incidental learning conditions compared to those in the P-SOD group (Burte & Montello, 2017).

### 1.3. Gender, Strategy Use, and Spatial Ability

Numerous studies have examined gender differences in spatial abilities, consistently finding that men tend to outperform women in large-scale spatial tasks (Ishikawa & Montello, 2006; Munion et al., 2019; Padilla et al., 2017; Peer et al., 2021). However, not all men consistently demonstrate superior spatial ability compared to women in real-world settings, suggesting that gender alone does not fully account for individual differences in spatial ability.

Beyond gender, differences in spatial strategies contribute significantly to individual variability. Research indicates that men and women often adopt different strategies when navigating (Lawton, 1994; Fernandez-Baizan et al., 2019; Pazzaglia & De Beni, 2001). Women tend to rely more on landmark-based or route-based (egocentric) strategies, which emphasize sequences of actions or turns linked to specific environmental cues. In contrast, men more frequently adopt survey-based (allocentric) strategies, which involve constructing a mental map of the environment that allows for flexible navigation and direct shortcutting. These survey-based strategies have been associated with higher levels of spatial accuracy and performance, especially in tasks requiring mental rotation, direction estimation, or flexible route planning (Münzer et al., 2006). Moreover, strategy use appears to interact with task demands: allocentric strategies are more advantageous in tasks requiring map-like understanding or spatial updating, whereas egocentric strategies may suffice for habitual or linear routes (Wolbers & Hegarty, 2010). Moreover, these individuals with a good sense of direction (SOD) are often capable of flexibly employing one or more spatial strategies when navigating complex environments (Weisberg et al., 2014). It is important to note, however, that strategy preference is not fixed by gender. Some women successfully adopt survey strategies and perform at levels comparable to men. Conversely, some men rely primarily on landmark cues. These findings suggest that spatial strategy use may be shaped by experience, training, or environmental familiarity rather than biological sex alone (Boone et al., 2018; Coluccia & Louse, 2004).

### 1.4. Effects of Navigation Modalities on Spatial Learning

Traditional and digital navigation aids have diverse effects on spatial cognition. Paper maps offer allocentric information but require mental transformations, increasing cognitive load (Ishikawa et al., 2008; Sholl, 1988; Roskos-Ewoldsen et al., 1998).

Modern navigation systems have become indispensable in everyday life, providing efficient guidance through visual, audio, or multimodal cues. However, a growing body of research suggests that such systems may negatively impact spatial learning. For instance, visual navigation aids like electronic maps and GPS interfaces, although highly practical, have been shown to reduce users’ acquisition of spatial knowledge compared to direct exploration (Fenech et al., 2010; Gardony et al., 2013; Gardony et al., 2015; Hejtmánek et al., 2018). This phenomenon, often referred to as visual distraction, occurs when visual instructions overload cognitive resources and impede the formation of accurate mental representations of space (Duif et al., 2020; Rochais et al., 2017).

In contrast, directly experiencing an environment through navigation allows for the development of more robust topological and directional knowledge (Ishikawa et al., 2008). Verbal navigation instructions have been proposed as another classic method. Studies found that audio cues can enhance route efficiency and reduce cognitive load under certain conditions (Gardony et al., 2013; Montello et al., 2004). However, audio instructions can still lead to reduced landmark recognition or spatial recall (Gardony et al., 2015).

In previous research, individuals with differing SOD levels responded differently to different types of information. For instance, G-SOD individuals tend to experience greater disruption from visuospatial interference tasks, while P-SOD individuals are more affected by verbal distractions (Garden et al., 2002). In related research, Wen et al. (2011) found that visual information impaired performance in P-SOD participants more than in G-SOD individuals during spatial tasks, such as route choice and map sketching. These findings suggest that individual differences in spatial ability modulate how navigation information—particularly visual and verbal cues—affect spatial learning.

In summary, visual and audio navigation tools, while designed to facilitate wayfinding, can interfere with spatial knowledge acquisition depending on the user’s spatial ability and the modality of the guidance. Understanding this interaction is crucial for the design of adaptive navigation systems that promote both efficiency and spatial learning.

### 1.5. Present Study

This study aims to investigate how individuals with good and poor SOD perform under different navigation modalities (visual, audio, and audio–visual). Prior research has shown that external navigation aids, such as visual or audio instructions, may inadvertently impair spatial learning by directing attention away from environmental features (Gardony et al., 2013; Hejtmánek et al., 2018). A growing body of research suggests that overreliance on external aids can impair the natural acquisition of environmental knowledge, particularly when such tools provide spatially aligned or turn-by-turn instructions that reduce the need for active spatial processing (Ishikawa et al., 2008; Münzer et al., 2006). However, such aids also improve navigational efficiency, making them indispensable for effective wayfinding.

Importantly, unlike traditional dual-task paradigms that introduce secondary, task-irrelevant interference (e.g., verbal memory loads), the present study embeds task relevant navigation information (visual, audio, and audio–visual guidance) directly into the learning environment. Rather than testing divided attention, the goal is to examine how the modality of helpful navigational cues influences spatial memory formation and how this effect is moderated by individual differences in SOD ability.

Specifically, we assess participants’ acquisition of landmark and route knowledge through a set of navigation memory tasks. If audio navigation facilitates spatial learning better than visual guidance, this would suggest that the visual distraction effects observed in earlier studies are not simply the result of divided attention but are possibly due to modality-specific processing limitations. Furthermore, it remains unclear whether individuals with a good or poor sense of direction (G-SOD or P-SOD) benefit differently from various navigation modalities. If navigation aids are shown to facilitate the acquisition of specific types of spatial knowledge, future systems could be optimized to accommodate individual differences, such as tailoring navigation modes to users with varying levels of SOD.

Previous studies have demonstrated the effectiveness of using driving simulators and desktop environments to study spatial memory (Gardony et al., 2013; Gardony et al., 2015; Seminati et al., 2022). Therefore, in this study, we also considered leveraging the virtual desktop environment to comprehensively compare and explore participants’ acquisition of spatial knowledge (landmarks, routes) by observing their performance in individual tasks.

## 2. Materials and Methods

### 2.1. Participants

Participants were first pre-screened from university students to select individuals who met the criteria for participation in the main study. A total of 708 copies of the Santa Barbara Sense of Direction (SBSOD) Scale (Hegarty et al., 2002) were distributed online to assess participants’ spatial ability, resulting in 278 male and 430 female valid responses. A total of 106 participants scoring in the top 20% were classified into the good spatial ability (G-SOD) group, while 142 participants scoring in the bottom 20% were classified into the poor spatial ability (P-SOD) group.

From the 106 G-SOD and 142 P-SOD participants selected, we contacted them to participate in the experiment. In the end, only 78 participants agreed to participate in the experiment. However, three participants withdrew midway through the experiment. Due to resource limitations, we were ultimately able to collect complete data from 75 participants. A post hoc power analysis using G*Power (Version 3.1; Faul et al., 2007) indicated that this sample size allows us to find effect sizes of at least f = 0.37 given alpha = 0.05 and a power of 0.80. There were 39 people with good spatial ability(G-SOD), 18 men and 21 women, *M*_SBSOD_ = 5.94, *SD*_SBSOD_ = 0.41; 36 people with poor spatial ability (P-SOD), 15 males and 21 females, *M*_SBSOD_ = 2.72, *SD*_SBSOD_ = 0.53. The average age of participants in the G-SOD group was 20.03 years (*SD* = 1.16), and in the P-SOD group, it was 19.89 years (*SD* = 0.85). There was no significant age difference between the two groups. The participants had normal or corrected normal vision, no red-green color blindness. They had signed the informed consent form before the experiment, and received certain reward after the experiment was completed.

### 2.2. Design

The present study employed a 2 × 3 factorial between-participants design, crossing spatial ability group (G-SOD, P-SOD) and navigation mode (visual, audio, audio–visual), resulting in six experimental conditions: G-SOD visual condition, G-SOD audio condition, G-SOD audio–visual condition, P-SOD visual condition, P-SOD audio condition, and P-SOD audio–visual condition. The dependent variables are the total number of correct responses in route tracing, correct responses in turn intersection, correct responses in recognizing scenes, and correct responses in recognizing the order.

### 2.3. Materials

#### 2.3.1. Pre-Screening Scale

The Santa Barbara Sense of Direction (SBSOD) Scale (Hegarty et al., 2002) was used to classify participants’ spatial ability levels. This 15-item self-report scale assesses individuals’ perceived navigational and directional capabilities. Participants rate their agreement with each item on a 7-point Likert scale ranging from “strongly disagree” to “strongly agree.” Higher scores reflect a better perceived sense of direction. The scale has demonstrated good psychometric properties across studies in spatial cognition.

#### 2.3.2. Learning Phase

**Figure 1:**
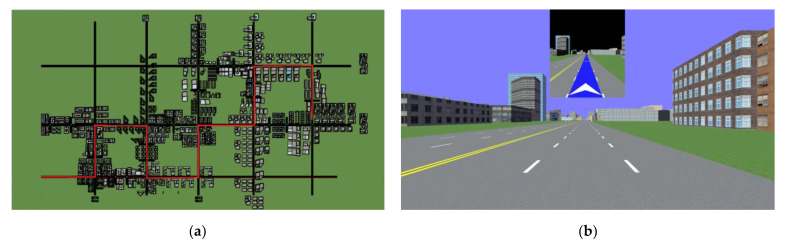
Examples of the experimental condition. (**a**) the scene map (the learning route is marked in red); (**b**) an example of the navigation interface. The navigation interface was displayed in the upper central part of the screen. The route that the participant needed to follow was indicated by a blue line. The white arrow within the interface signified that the driving direction should be kept straight ahead.

The experimental scenario utilized a virtual city road model constructed using SketchUp 2016 software. It featured three horizontal roads and five vertical roads, each with a width of 3.5 m. The total length of the route that participants needed to learn during the experiment was approximately 8 km. The road consisted of 9 turn intersections and 16 straight intersections. A certain number of landmarks were set for each route, included buildings and markers, etc., which were evenly distributed on both sides of the road. To ensure clear distinction, we have also labeled landmarks with specific names, such as Fair Station, Bookstore, etc. This enabled participants to encounter various named landmarks during their learning phase, ensuring consistent reference points and a uniform understanding of the buildings. Refer to Figure 1a for the scene map and Figure 1b for an example of the navigation interface.

The virtual scene was programmed using Vizard software version 5.0 and displayed on screen. Participants navigated the virtual environment on a 22-inch Widescreen LCD monitor at 1920 × 1080 resolution with a simulated field of view of 90° and sat at a viewing distance of approximately 50 cm. The virtual scene was presented as depicted in Figure 1b. Participants in the experiment controlled the steering using a simulated steering wheel, with configured foot pedals enabling speed control. Once the participant started to simulate driving in the virtual environment, visual navigation came into play, which provided the participants with information on the route they needed to follow ahead of their current position. The route which the subject needed to navigate was marked by a blue line. If the intersection ahead is kept straight or a turn is required, the navigation interface would present the area around the intersection ahead and show a white straight or turning arrow at the intersection in the navigation interface before reaching the location of the intersection. The visual navigation interface always indicated a dynamic representation of the route to be followed ahead of the current position, so it changed constantly with the movement of the simulated driving position. Audio guidance used the traditional “turn left/go straight/turn right at the intersection ahead” prompt. The program monitored the participant’s position in a real-time manner and activated a voice to prompt the participant to turn left/go straight/turn right before reaching the intersection where the decision needed to be made. The audio–visual navigation mode is a combination of the first two, participants could obtain both visual and audio information about the route to follow ahead (consistent information in two types). In the visual and audio–visual conditions, the navigation interface was displayed in the upper central part of the screen, which did not obstruct the view of the presented scene. Therefore, participants were able to see the complete visual scene, and the navigation cues (arrows or instructions) did not cover any crucial visual information within the environment. Under the audio-only condition, participants only received audio cues without any visual guidance. This ensures that the differences observed between the audio and other conditions are not due to obstruction or differences in the presentation of the scene maps but rather the modality of the navigation cues (visual, audio, or combined audio–visual). Visual/audio/audio–visual navigation was stopped until the end point was reached. All three types of guidance were egocentric in their navigation cues.

#### 2.3.3. Test Phase

Spatial memory was assessed with three tasks.

Route retracing: The route retracing task involved participants navigating the same environment as in the learning phase, but without the aid of navigation information (visual, audio, or audio–visual). They were required to drive to the endpoint based solely on their memory of the route. In case of errors at intersections, the program automatically corrected their current position to the correct one, allowing them to continue driving forward without needing to backtrack to the intersection for a new decision. During this phase, the program recorded any errors made at intersections. Participants had to rely on their memory to navigate the route, making decisions at intersections and replicating the route from the learning phase in terms of both distance and direction. This phase primarily assessed the participants’ ability to recall and apply route knowledge acquired during the learning phase.

The route retracing task required participants to reproduce the learned route by identifying the correct direction at intersections. The route included two types of intersections, namely turn intersections (where participants had to actively make a directional choice—left or right) and straight intersections (requiring no directional change). To capture both overall performance and decision-making accuracy in more cognitively demanding situations, we computed two measures, which were (1) the total number of correctly identified intersections (including both straight and turn) and (2) the number of correct responses at turn intersections only. This distinction allows for a more fine-grained analysis of participants’ spatial decision-making, as turn points typically involve higher working memory load and more complex spatial encoding (e.g., direction updating and landmark association) and are thus considered more challenging (Ishikawa & Montello, 2006; Wiener et al., 2009).

Recognizing scenes: Recognizing scenes involved randomly presenting pictures of scenes on the computer screen, including scenes that appeared on the correct route and those that did not. Participants were required to determine whether each scene had appeared during the learning phase by pressing the F key for “yes” or the J key for “no”. The program recorded participants’ reaction times and their specific key responses. A total of 28 images were presented for judgment, half of which had appeared during the learning phase and half that had not. This stage primarily assessed participants’ knowledge of landmarks, as the images that required them to make F judgements were actual landmarks from the scenes. This task would be easy for the participants if they had learned about the landmarks during the learning phase.

Recognizing the order: The computer screen displayed two pictures of scenes that had appeared during the learning phase and asked participants to judge their order: pressing the F key if the scene on the left appeared first, or the J key otherwise. Making the correct judgment indicated that participants had grasped the distance and sequence between the two landmarks, extending their knowledge from specific landmarks to the entire route. This task enabled the measurement of participants’ route knowledge. The program recorded participants’ reaction times and their specific key responses. A total of 28 pictures were presented for judgment, with half of the scenes appearing first on the left and the other half on the right.

### 2.4. Procedure

Before the experiment commenced, participants completed the virtual navigation and navigational aid training. In the training phase, participants were instructed to simulate driving to ensure consistent perspectives maintain scientific validity across all participants’ operations. They familiarized themselves with simulated driving procedures and navigation mode presentations. Driving behaviors adhered to traffic regulations, such as driving in the middle lane whenever possible, and avoiding crossing the yellow line. Scenes during the training phase were intentionally different from those in the learning phase to prevent interference with post-learning tasks, specifically by avoiding similar or identical landmarks.

The experimental session adhered to training requirements, where participants engaged in simulated driving under visual, audio, or combined audio–visual navigation conditions. Additionally, participants were instructed to memorize the environment they would navigate. The route was learned twice, with the second session starting immediately after completing the first. Following the learning phase, participants immediately began the test phase, without any intervening time or additional requirements. During the test phase, participants were required to complete three tasks, route retracing, recognizing scenes, recognizing the order. The entire experiment lasted approximately 55 min.

## 3. Results

Due to the particular character of our grouping, we did not achieve complete gender balance across navigation mode conditions during data collection. As per our research objectives, we included SOD ability and navigation mode as independent variables in our analysis, with route-retracing correct number of intersections, route-retracing turn intersection correct number, correct responses in recognizing scenes and recognizing the order as dependent variables. Initially, we conducted independent samples t-tests to compare groups by gender, as shown in Table 1. The results indicated that gender did not have a statistically significant effect on any of the dependent variables (all *p* > 0.05). Therefore, gender was not included in further analyses.

**Figure 2:**
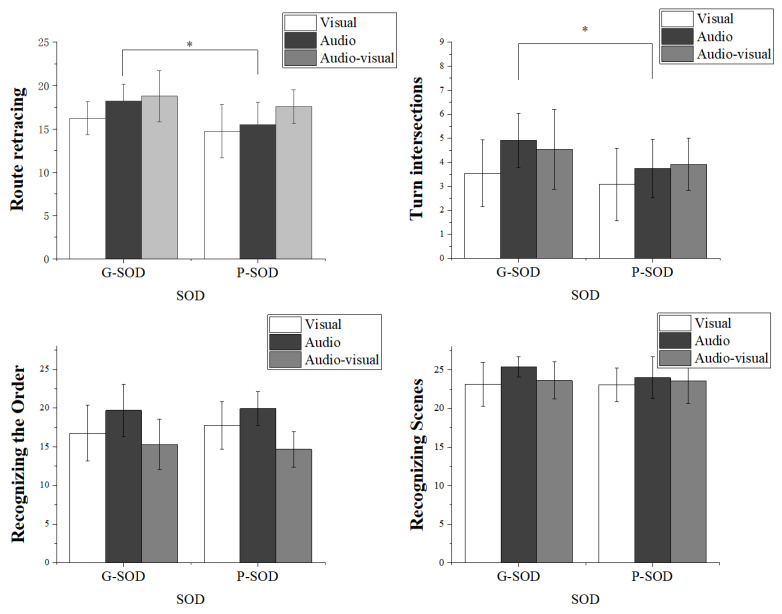
Performance of participants on each behavioral indicator across three tasks, illustrating the main effect of SOD. Each subplot represents a different dependent variable: route retracing (total correct number), turn intersections (correct number), recognizing scenes (correct number), and recognizing the order (correct number). Bars represent mean scores for G-SOD and P-SOD participants under visual, audio, and audio–visual navigation conditions. Error bars indicate standard deviations. (Notes. * indicates *p* < 0.05).

**Figure 3:**
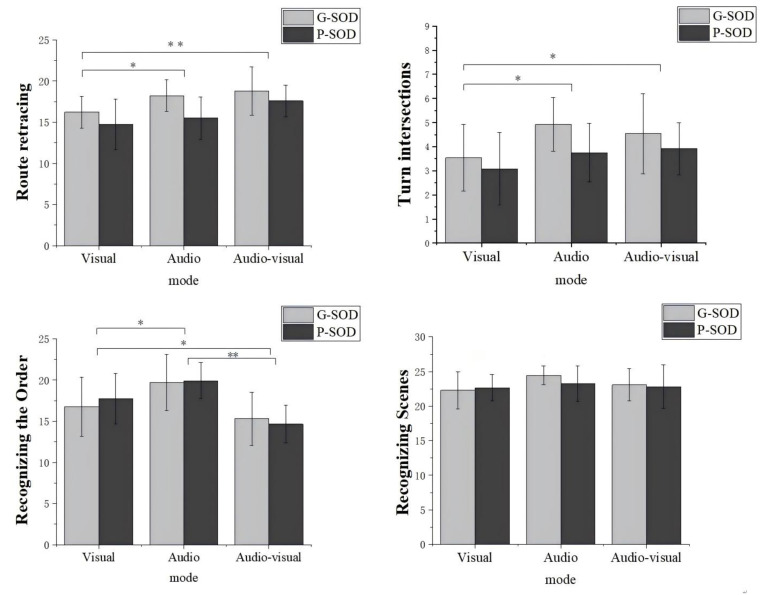
Performance of participants on each behavioral indicator across three tasks, illustrating the main effect of navigation mode. Each subplot represents a different dependent variable: route retracing (total correct number), turn intersections (correct number), recognizing scenes (correct number), and recognizing the order (correct number). Bar graphs display the mean performance scores under visual, audio, and audio–visual modes for each task. Error bars represent standard deviations.(Notes. * indicates *p* < 0.05, ** indicates *p* < 0.01).

A 2 SOD group (G-SOD, P-SOD) × 3 navigation mode (visual, audio, audio–visual) multivariate ANOVA was run on the four dependent measures together: route-retracing correct number of intersections, route-retracing turn intersection correct number, recognizing scenes (numbers of correct key press) and recognizing the order (numbers of correct key press). The results indicated a significant effect of SOD group, *p* = 0.029 < 0.05, η^2^ = 0.148; as well as a significant effect of navigation mode, *p* = 0.000 < 0.05, η^2^ = 0.298. No interaction effect between the two factors was observed, *p* = 0.861 > 0.05, η^2^ = 0.028. Detailed results are illustrated in Figure 2 and Figure 3. When differences in reaction times between the six groups were calculated, the results indicated that there were no significant differences in three tasks. To explore these results more closely, we next look at each measure of spatial memory task individually.

Route retracing task.

A 2 SOD group (G-SOD, P-SOD) × 3 navigation mode (visual, audio, audio–visual) ANOVA was conducted on route retracing with the total correct number of intersections. The results revealed a significant main effect of the SOD group, *F*(1, 69) = 10.171, *p* = 0.002, η^2^ = 0.128. The G-SOD group showed a higher total correct number of intersections (*M* = 17.74, *SD* = 0.39) compared to the P-SOD group (*M* = 15.94, *SD* = 0.41). The main effect of navigation mode was significant, *F*(2, 69) = 7.557, *p* = 0.001, η^2^ = 0.180. Participants in the audio–visual navigation mode performed the best (*M* = 18.18, *SD* = 0.49), followed by the audio navigation mode (*M* = 16.87, *SD* = 0.49) and the visual navigation mode (*M* = 15.49, *SD* = 0.49). The difference between the visual and audio conditions was significant (*p* = 0.046). The difference between the visual and audio–visual conditions was significant (*p* = 0.000). There was a marginal difference between the audio and audio–visual modes, *p* = 0.068. The interaction of the SOD group and navigation mode were not significant, *F*(2, 69) = 0.705, *p* = 0.498, η^2^ = 0.020.

In terms of turn intersection correct numbers in route retracing, a 2 SOD group (G-SOD, P-SOD) × 3 navigation mode (visual, audio, audio–visual) ANOVA was conducted. The results revealed a significant main effect of the SOD group, *F*(1, 69) = 5.791, *p* = 0.019, η^2^ = 0.077. The G-SOD group (*M* = 4.33, *SD* = 0.22) performed better than the P-SOD group (*M* = 3.58, *SD* = 0.23). The main effect of navigation mode was significant, *F*(2, 69) = 4.356, *p* = 0.017, η^2^ = 0.112. The audio navigation mode (*M* = 4.34, *SD* = 0.27) and the audio–visual navigation mode (*M* = 4.23, *SD* = 0.27) both outperformed the visual navigation mode (*M* = 3.31, *SD* = 0.27), with *p* = 0.008 and *p* = 0.001, respectively. The interaction between the SOD group and navigation mode was not significant, *F*(2, 69) = 0.485, *p* = 0.618, η^2^ = 0.014.

Recognizing scenes task

A 2 SOD group (G-SOD, P-SOD) × 3 navigation mode (visual, audio, audio–visual) ANOVA was conducted on recognizing scenes. The analysis revealed no significant main effect for the SOD group, *F*(1, 69) = 0.438, *p* = 0.510, η^2^ = 0.006. The result suggested that participants with a good sense of direction (G-SOD) (*M* = 23.28, *SD* = 0.38) did not perform significantly differently from those with a poor sense of direction (P-SOD) (*M* = 22.92, *SD* = 0.40) in recognizing scenes. Similarly, the main effect of navigation mode was not significant, *F*(2, 69) = 2.118, *p* = 0.128, η^2^ = 0.058, indicating that the type of navigation mode (visual, audio, or audio–visual) did not significantly influence the accuracy of scene recognition. Specifically, the mean number of correct responses was *M* = 22.49, *SD* = 0.48 in the visual mode, *M* = 23.86, *SD* = 0.48 in the audio mode, and *M* = 22.96, *SD* = 0.48 in the audio–visual mode. Furthermore, the interaction between the SOD group and navigation mode was not significant, *F*(2, 69) = 0.687, *p* = 0.507, η^2^ = 0.020, suggesting that the effects of navigation mode on scene recognition did not vary significantly across different levels of SOD ability. In other words, both G-SOD and P-SOD participants demonstrated similar performance across the three navigation modes.

Recognizing the order task

A 2 SOD group (G-SOD, P-SOD) × 3 navigation mode (visual, audio, audio–visual) ANOVA was conducted on recognizing the order. The analysis revealed no significant main effect for the SOD group, *F*(1, 69) = 0.072, *p* = 0.789, η^2^ = 0.001. The result suggested that participants with a good sense of direction (G-SOD) (*M* = 17.26, *SD* = 0.49) did not perform significantly differently from those with a poor sense of direction (P-SOD) (*M* = 17.44, *SD* = 0.51) in recognizing the order. A significant main effect of navigation mode was observed, *F*(2, 69) = 15.785, *p* = 0.000, η^2^ = 0.314. Specifically, participants in the audio mode demonstrated the highest number of correct responses, (*M* = 19.80, *SD* = 0.61), significantly outperforming those in the visual mode (*M* = 17.26, *SD* = 0.61, *p* = 0.004) and the audio–visual mode (*M* = 14.99, *SD* = 0.61, *p* = 0.000). Additionally, participants in the visual mode outperformed those in the audio–visual mode, with a significant difference (*p* = 0.011). These results suggested that audio guidance, when provided as the sole navigation mode, was the most effective in improving participants’ ability to correctly recognize the sequence of scenes and actions, followed by visual navigation and then the combined audio–visual navigation mode. Furthermore, the interaction between the SOD group and navigation mode was not significant, *F*(2, 69) = 0.447, *p* = 0.641, η^2^ = 0.013. The details of the descriptive statistics are presented in Table 2.

## 4. Discussion

The present study investigated how different navigation modalities (visual, audio, and combined audio–visual) affect spatial navigation efficiency, particularly in individuals with varying levels of spatial ability, categorized as G-SOD (good sense of direction) and P-SOD (poor sense of direction). Rather than comparing the presence versus absence of navigational aids, our focus was on examining the relative effectiveness of each navigation mode. The results revealed clear and meaningful main effects: both spatial ability and navigation mode independently influenced performance on spatial tasks. However, no significant interaction was found between spatial ability and navigation mode, indicating that the impact of each navigation type was consistent across individuals with different spatial abilities. These findings suggest that while both individual spatial ability and the type of navigational information play important roles in spatial task performance, their effects may operate independently rather than interactively. G-SOD participants outperformed P-SOD participants in route retracing task, both in total intersection accuracy and in turn intersection accuracy. Audio navigation mode led to better performance in route retracing (turn intersection) and recognizing the order tasks. No SOD group differences were observed between recognizing scenes and recognizing the order tasks. No navigation mode differences were observed in the recognizing scenes task. These findings highlight the differential benefits of navigation modes based on individual spatial ability, with implications for designing personalized navigation systems.

These findings are generally consistent with previous studies that have employed spatial ability as an independent variable, wherein participants with good spatial ability (G-SOD) consistently outperformed those with poor spatial ability (P-SOD) on spatial memory tasks (Burte & Montello, 2017; Wen et al., 2011; Wen et al., 2013). According to the three stages of spatial knowledge acquisition proposed by Siegel and White (1975), the tasks selected in our study effectively captured distinct levels of spatial knowledge: landmark and route knowledge. G-SOD participants exhibited better overall performance across all four dependent measures, statistically significant differences emerged primarily in tasks assessing route knowledge. This aligns with findings from previous research and daily observations of superior wayfinding abilities among individuals with G-SOD (Burte & Montello, 2017; Wen et al., 2011; Wen et al., 2013).

These results corroborate the findings of Wen et al. (2013), who suggested that individuals with G-SOD possess a clear advantage in developing route knowledge of the environment. They are more capable of utilizing information from multiple sources and effectively encoding and integrating this information into coherent spatial representations, in contrast to individuals with P-SOD. The results of our experiments also supported this conclusion. In contrast, no significant group differences were found in tasks assessing landmark knowledge. This may be due to the relatively low cognitive demands of such tasks, which rely less on directional or distance information and are typically remembered more easily (Siegel & White, 1975). According to Siegel and White’s (1975) framework, this suggests that group differences become more apparent when tasks tap into higher-level spatial knowledge. The recognizing scenes task, which measured this basic level of spatial knowledge, likely did not challenge participants sufficiently to reveal differences between the groups.

Furthermore, no significant group differences were observed in recognizing scenes and recognizing the order task between G-SOD and P-SOD participants. This outcome may be attributed to the nature of the task itself. Successful completion of recognizing the order task required participants to first accurately encode and retain the sequence of multiple landmarks encountered during navigation. As previously discussed, there were no significant differences in landmark knowledge acquisition between the two groups, suggesting that both G-SOD and P-SOD individuals were similarly proficient in recognizing individual landmarks. Given this shared foundation, it is likely that participants from both groups were equally capable of forming basic sequential associations between landmarks. Moreover, because the task was carefully designed with strict control over the sequence of landmark presentations, all participants were required to make judgments based on the same set of paired comparisons. This methodological control likely minimized variability related to individual strategies or recall biases, leading to uniform performance across groups.

The effect of navigation mode on spatial task performance was evident, as participants in the audio navigation mode outperformed those in the visual-only and combined audio–visual modes on both the route retracing task (turn intersection) and recognizing the order task. Specifically, participants guided solely by audio cues demonstrated significantly higher accuracy in the decision-making of turning points. This suggested that audio navigation is more effective than visual or audio–visual navigation in guiding participants through complex spatial decisions, such as making turns. Turning decisions typically require individuals to rapidly integrate multiple types of spatial information, including changes in direction, landmark recognition, and route updating, which imposes a high cognitive load (Ishikawa & Montello, 2006; Wiener et al., 2009). This superior performance in audio navigation mode may be attributed to several factors. First, when navigational information is delivered through audio cues, visual attention can remain focused on the environment itself, reducing visual interference and thereby improving the accuracy of turning point judgments. One plausible explanation lies in the concept of visual distraction. The visual navigation interface demands considerable attentional resources, as users must not only process visual cues but also relate them to the surrounding environment to guide movement (Hejtmánek et al., 2018; von Stülpnagel & Steffens, 2013). This continuous visual processing may interfere with the encoding of environmental features into memory. In contrast, audio navigation requires no perspective shift or visual transformation (Ishikawa et al., 2008), allowing participants to follow route instructions without diverting their visual attention away from the environment. Second, audio cues align more closely with the mechanisms of verbal working memory, enabling more efficient encoding of spatial information while preserving visual attention for environmental scanning. As such, the audio navigation mode may facilitate better integration of spatial cues by reducing cognitive load and enhancing dual-channel processing—supporting both verbal instruction and visual perception. This finding aligns with previous research, which suggests that both verbal and spatial working memory components play a critical role in encoding the environment and forming accurate spatial representations (Garden et al., 2002; Seminati et al., 2022). Audio navigation cues, in particular, may reduce visual processing demands, allowing participants to allocate more cognitive resources toward building. Third, the consistency between the visual input during learning and testing in audio navigation mode, where no overlay interface was present, may have facilitated a more effective encoding and retrieval of scene elements. In contrast, participants in the visual and audio–visual navigation modes were guided by a visual interface that overlaid part of the scene. This could have occluded peripheral visual information that was later fully visible during the testing phase, potentially introducing an encoding–retrieval mismatch. This mismatch may have reduced participants’ task performance.

On the other hand, earlier studies have demonstrated that multisensory feedback, integrating information from multiple sensory channels, can enhance navigation performance (Ernst & Bülthoff, 2004; Jones et al., 2008). Our results provide further empirical support for this claim. Specifically, in the route retracing task (total intersection accuracy), participants in the combined audio–visual navigation mode achieved a higher rate of correct responses compared to those in either the visual-only or audio-only modes. This suggests that the integration of audio and visual cues may support the formation of more robust spatial representations by reinforcing information through multiple modalities. These findings collectively underscore the potential advantages of multisensory navigation aids, particularly for tasks requiring comprehensive spatial recall.

For participants who needed to acquire route knowledge, the task could be better accomplished based on audio information. The additional visual interface not only attracted the individual’s attention but also added information load to them. They needed to assess whether information from different sources is consistent, and even overloads could led them a lower propensity for intentional memory.

Though all of these results were in accordance with the existing literature, previous findings had also found that G-SOD and P-SOD participants were disturbed by different kinds of information, respectively. However, we cannot conclude from the results of this study which navigation information was preferred by participants with different spatial abilities or found a more suitable navigation mode for different groups. From analysis of the results, the total number of correct retracing intersections for both G-SOD and P-SOD subjects seemed to maintain an increasing trend in all three navigation modes (visual<audio<audio–visual), however the magnitude of the growth (gradient) and the respective preferred navigation patterns seemed to differ between modes. For G-SOD individuals, the average number of correct intersections in both the audio and combined audio–visual modes were better than the average number of correct intersections for participants in the visual condition. Though the standard deviation in the combined audio–visual mode was larger than the other two conditions, to some extent it still reflected the preference of some participants. For P-SOD individual, the number of correct intersections in combined audio–visual mode was higher than the other two navigation modes and had a smaller standard deviation. Evidence, unfortunately, was limited, our results did not show a statistically significant interaction effect between SOD groups and navigation mode. A possible explanation for this result may lie in two key factors.

The first factor is task selection. Previous studies that have drawn conclusions have used dual-task paradigm, usually asked participants to learn the primary task while also asked them to pay attention to a secondary task that contains distracting information (Garden et al., 2002; Meilinger et al., 2008; von Stülpnagel & Steffens, 2013; Wen et al., 2013). The primary and secondary tasks belonged to different categories and were independent of each other, so the participants’ attention resources were allocated to the tasks in a opposite relationship. In the present study, navigational information distracted participants to some extent. However, the information itself was intended to assist navigation by helping participants remember the environment and complete the tasks during the test phase, even if some of their attention was diverted to processing the guidance. On the other hand, previous tasks measured correlations of short-term or working memory, and participants placed more emphasis on visual spatial resources when learning routes from maps, whereas tasks in a virtual or real environment could mobilize cues of a different nature. The present study primarily focused on assessing route and landmark knowledge. Although previous research suggests that survey-level knowledge may emerge incidentally during route learning (Spiers & Maguire, 2008; Wiener et al., 2009), our experimental design did not include a pointing (relative direction judgment) task, which is considered more effective for assessing survey-level spatial knowledge. Future research could perhaps include a task to measure survey-level spatial knowledge. The advantages of audio or visual navigation in wayfinding behavior might be related to daily habits, cognitive load, and other factors, which could be further explored later.

The second factor is personal strategies and preferences. As noted in a study by Hejtmánek et al. (2018), the conclusions suggested that impaired spatial knowledge may be driven in part by personal strategies and preferences, and that P-SOD people more suspect their skills were therefore more dependent on GPS. If this is the case, then participants with P-SOD should be more influenced by navigation, so their worse performance is not necessarily due to their own poor spatial ability.

Despite the valuable findings, several limitations should be acknowledged. First, our sample size was constrained by practical limitations; the observed effects were of moderate-to-large size. The relatively small sample size may still limit the generalizability of the results. Second, the relatively narrow age range of participants, primarily college students, may limit the generalizability of the findings. Third, the spatial tasks used in this study primarily assessed landmark and route knowledge. Although some of the literature suggests that survey knowledge may gradually develop alongside route learning (Ishikawa & Montello, 2006), our experiment did not include a pointing task or relative direction judgment task, which are more sensitive measures of survey-level spatial understanding. Fourth, while we used the Santa Barbara Sense of Direction (SBSOD) scale to classify participants into G-SOD and P-SOD groups, we did not perform item-level analyses. This limits our ability to understand which specific aspects of self-reported spatial ability (e.g., directional estimation, mental mapping, or orientation in unfamiliar environments) are most predictive of spatial task performance.

While the present study provides valuable insights into the effects of navigation mode and individual spatial abilities on spatial memory acquisition, several avenues for future research remain open. First, habitual reliance on GPS has been shown to reduce spatial learning and cognitive map development (Gardony et al., 2013; Gardony et al., 2015; Hejtmánek et al., 2018). Future studies could further explore how GPS dependency, individual navigation strategies (Pazzaglia & De Beni, 2001), and spatial confidence (Hegarty et al., 2002) affect performance under different navigation modes, particularly for P-SOD individuals, who may be more dependent on external aids. Second, future studies could test these effects in real-world or immersive VR environments, which more closely mimic natural navigation contexts and may engage different cognitive and sensorimotor processes than desktop-based VR simulations (Riecke et al., 2007; Spiers & Maguire, 2008; Chrastil & Warren, 2012). Third, future studies with larger and more diverse samples are encouraged to validate and extend the current findings. Prior studies have shown that age-related decline (Moffat, 2009) and age-related differences in navigation strategies (Yamamoto & DeGirolamo, 2012) can all influence spatial cognition. Therefore, incorporating these factors will enable more robust conclusions. Fourth, future studies could include a relative direction judgment task (Lei & Mou, 2022; Liu et al., 2012), which is a more sensitive measure of survey knowledge. This addition would allow for a more comprehensive assessment of how different navigation modalities influence the acquisition of landmark, route, and survey knowledge. Fifth, future research could explore item-level patterns to identify more nuanced links between individual spatial traits and navigation outcomes (Hegarty et al., 2002).

## 5. Conclusions

This study reveals how different navigation modalities (visual, audio, and audio–visual) and individual spatial ability (as indexed by SOD) influence the acquisition of spatial knowledge. Participants with a good sense of direction (G-SOD) outperformed those with a poor sense of direction (P-SOD) in the route retracing task. Audio navigation mode proved to be particularly effective in supporting accurate spatial decision-making, especially at turn intersections, where cognitive demands are higher. Audio navigation mode also proved to be particularly effective in recognizing the order task. Participants in audio navigation mode showed better performance than those in visual or audio–visual modes, suggesting that audio instructions may reduce visual distraction and support more efficient environmental encoding. The absence of significant interaction effects between the SOD group and navigation mode indicates that the benefits of audio guidance apply broadly, regardless of spatial ability level. However, the scene recognition task did not yield significant differences, possibly due to ceiling effects or the limited complexity of visual memory demands. These findings highlight the importance of tailoring navigation support to cognitive profiles and inform the design of more adaptive navigation systems.

## Figures and Tables

**Table 1:** Effect of gender on dependent variables.

	Male (n = 33)		Female (n = 42)		*t*	*p*
	*M*	*SD*	*M*	*SD*		
Route retracing	17.36	2.40	16.5	3.01	1.345	0.183
turn intersection	4.00	1.20	3.95	1.62	0.141	0.888
Recognizing Scenes	24.15	2.06	23.55	2.76	1.209	0.298
Recognizing the Order	17.30	3.57	17.38	3.58	−0.094	0.926
Route retracing RT (ms)	546,213.33	50,100.94	541,563.71	46,479.63	0.416	0.782
Recognizing Scenes RT (ms)	2029.30	494.60	1948.56	363.35	0.815	0.069
Recognizing the Order RT (ms)	3485.72	856.83	3162.76	821.16	1.659	0.683

**Table 2:** Means and standard deviations of the route retracing total correct number, turn intersection correct number, recognizing scenes correct number, recognizing the order correct number in three tasks.

		G-SOD (n = 39)			P-SOD (n = 36)		
		Visual (n = 13)	Audio (n = 13)	Audio–Visual (n = 13)	Visual (n = 12)	Audio (n = 12)	Audio–Visual (n = 12)
Route retracing	Total CN (25)	16.23 (1.92)	18.23 (1.92)	18.77 (2.95)	14.75 (3.08)	15.50 (2.58)	17.58 (1.93)
turn intersection	CN (9)	3.54 (1.39)	4.92 (1.12)	4.54 (1.66)	3.08 (1.51)	3.75 (1.22)	3.92 (1.08)
Recognizing Scenes	CN (28)	23.15 (2.83)	25.38 (1.33)	23.62 (2.40)	23.08 (2.19)	24.00 (2.66)	23.58 (2.91)
Recognizing the Order	CN (28)	16.77 (3.61)	19.69 (3.40)	15.31 (3.25)	17.75 (3.05)	19.92 (2.19)	14.67 (2.27)

Note. CN = correct number.

## Data Availability

The datasets generated during and/or analyzed during the current study are available from the first author upon reasonable request.
